# Fabrication of Pre-Structured Substrates and Growth of CIGS Micro-Absorbers

**DOI:** 10.3390/nano14060543

**Published:** 2024-03-20

**Authors:** Marina Alves, Pedro Anacleto, Vasco Teixeira, Joaquim Carneiro, Sascha Sadewasser

**Affiliations:** 1 International Iberian Nanotechnology Laboratory, Av. Mestre José Veiga s/n, 4715-330 Braga, Portugal; marina.alves@inl.int (M.A.); pedro.anacleto@inl.int (P.A.); 2 Centre of Physics of Minho and Porto Universities (CF-UM-UP), Azurém Campus, University of Minho, 4800-058 Guimarães, Portugal; vasco@fisica.uminho.pt

**Keywords:** CIGS, thin-film photovoltaics, micro-concentrator photovoltaics, sputtering, micro solar cell

## Abstract

Second-generation thin-film Cu(In, Ga)Se_2_ (CIGS) solar cells are a well-established photovoltaic technology with a record power conversion efficiency of 23.6%. However, their reliance on critical raw materials, such as In and Ga, requires new approaches to reduce the amount of critical raw materials employed. The micro-concentrator concept involves the combination of thin-film photovoltaic technology with concentrator photovoltaic technology. This approach reduces the size of the solar cell to the micrometer range and uses optical concentration to collect sunlight from a larger area, focusing it onto micro solar cells. This work is devoted to the development of a process for manufacturing pre-structured substrates with regular arrays of holes with 200 and 250 µm diameters inside a SiO_x_ insulating matrix. Subsequently, a Cu–In–Ga precursor is deposited by sputtering, followed by photoresist lift-off and the application of a Cu–In–Ga thermal annealing at 500 °C to improve precursor quality and assess pre-structured substrate stability under elevated temperatures. Finally, a two-stage selenization process leads to the formation of CIGS absorber micro-dots. This study presents in detail the fabrication process and explores the feasibility of a bottom-up approach using pre-structured substrates, addressing challenges encountered during fabrication and providing insights for future improvements in CIGS absorber materials.

## 1. Introduction

From 2021 to 2022, global photovoltaic installations increased from 175 GW to 240 GW, an overall growth of 65 GW. China dominated the market, installing 106 GW in 2022, corresponding to 44% of the global market. The European Union followed, installing 38.7 GW, led by Spain (8.1 GW), Germany (7.5 GW), Poland (4.9 GW), and the Netherlands (3.9 GW) [1]. For 2023, analysts project a substantial increase, estimating that photovoltaic installations could reach approximately 440 GW, double that of 2022 [2]. In 2021, crystalline-silicon (c-Si) photovoltaics represented a 95% share of production [3]. This technology achieved record efficiencies of 26.8% for mono-crystalline, 24.4% for multi-crystalline, and 27.6% for concentrator mono-crystalline [4,5]. To further enhance efficiency, new PV cell architectures like PERC (Passivated Emitter and Rear Cell) [6] have been explored. The main difference from a typical monocrystalline silicon cell lies in the inclusion of a passivation layer (a dielectric material) on the back cell’s surface, promoting an increase in the efficiency of the PV solar cell. Thin-film solar cells have emerged as a compelling and cost-efficient alternative to c-Si. Recently, Keller et al. [7] reported a 23.6% record efficiency for non-concentrated (Ag,Cu)(In,Ga)Se_2_ solar cells by introducing Ag into the absorber, implementing a Ga profile with a high concentration of Ga close to the back contact and lower concentration closer to the CdS buffer layer, and a post-deposition treatment with RbF. Ward et al. [8] presented CIGS concentrator cells grown by physical evaporation using a modified three-stage process, achieving an efficiency of 20.8% under 1 sun and a record efficiency of 23.3% at 14.7 suns. Furthermore, CdTe solar cells have achieved an efficiency of 22.3% [5].

Concentrator photovoltaic (CPV) technology uses optical elements such as mirrors and/or lenses to concentrate light onto small and highly efficient solar cells [9], aiming to enhance overall performance. An ideal CPV system incorporates cost-efficient concentrating optics to reduce the active solar cell area, enabling the use of more expensive, high-efficiency solar cells [10]. Remarkable efficiencies of 47.6% at 665 suns [11] and 30.8% at 61 suns [12] have been achieved for four-junction (GaInP/GaInAs; GaInAsP/GaInAs) and flexible GaAs solar cells, respectively.

To enhance power conversion efficiency (PCE), reduce material usage, and lower costs, micro-concentrator photovoltaics (micro-CPVs) have been intensively investigated in recent years [10,13,14]. The micro-CPV concept consists of reducing the solar cells’ lateral dimensions to the range of hundreds of micrometers, resulting in cell areas below 1 mm^2^ [14,15]. Thin-film micro-CPVs present several advantages over conventional CPVs and other technologies, including enhanced cell performance, material savings, efficient thermal management, reduced series resistance losses, and thinner modules [16]. Through simulations, Sadewasser et al. [17] demonstrated that micro-concentrator solar cells offer a substantial advantage over regular CPV devices. The smaller size of devices leads to improved heat management, and for micro solar cells with a diameter of <200 µm, the temperature can be kept at less than 10 °C above the temperature of a flat panel solar cell. Paire et al. [18] showed that reducing cell size to the micrometric range allows better thermal evacuation due to a higher surface-to-volume ratio and reduced resistance losses. The microcells showed a 4% absolute improvement in efficiency at a concentration ratio of ×120, increasing from 13% to 17%. Under a concentration of ×1000, the microcells with a diameter under 50 µm had a temperature increase of less than 20 K.

In recent years, CIGS micro-CPVs have attracted increasing attention, and significant progress has been made, demonstrating the feasibility of the CIGS micro-concentrator concept. The required micro-CIGS solar cells can be fabricated using either a top-down or bottom-up approach. 

The top-down approach involves isolating the microcells within a large-area solar cell by shadowing, selective deposition, or isolation. Paire et al. [19] fabricated a device with a glass–Mo–CIGS–CdS–ZnO structure featuring a 15-micrometer-diameter active area, with an efficiency of 17% at ×120. The microcells were created by depositing a 400-nanometer-thick insulating SiO_2_ layer and a 20 nm/300 nm Ti/Au bilayer on top of the buffer layer. The holes in the SiO_2_ layer were defined by photolithography, after which the ZnO window layer was deposited. In 2013, Paire [20] prepared the CIGS absorber by co-evaporation, creating microcells with a 50-micrometer diameter using photolithography and achieving an efficiency of 21.3% at ×475. In a subsequent study [21], CIGS mesa microcells were fabricated through chemical etching. First, an array of patterned photoresist was coated on the cell surface. Then, the front window and CdS layers were etched with a hydrochloric acid solution. Finally, the CIGS layer was chemically etched with a bromine-based solution. The resulting devices exhibited an average absolute efficiency increase of 2% per concentration decade up to approximately 1000 suns. Reinhold et al. [22] developed lamellar-shaped micro solar cells with monolithic interconnects through standard P1, P2, and P3 scribes. This patterning allows a serial connection of single solar cells to form a solar module. The Mo back contact was divided with a P1-scribe, followed by a P2-scribe into the CIGS layer. Finally, a P3-scribe separates the front contact between two cells. The P1-scribe was made using a laser, while the P2 and P3 were mechanically scribed. The lamellar microcells can have an absolute 3.8% efficiency increase under concentrated white light. 

In the bottom-up approach, the absorber is locally grown in patterned/defined areas by using masks, patterned substrates, or pre-structured substrates, thereby reducing the usage of critical materials. While various methods can be used to deposit large-area CIGS absorbers, not all allow for local absorber growth. According to the literature, material-efficient deposition methods for CIGS or CuInSe_2_ (CIS) micro-absorbers include electrodeposition, physical vapor deposition (PVD), and laser-induced forward transfer. Ringleb et al. [23] demonstrated the growth of CIS and CIGS by nucleation of In droplets on laser-patterned substrates during physical vapor deposition and by laser-induced forward transfer (LIFT) of Cu, In, and Ga precursor layers. Efficiencies of 3.06% at 3 suns and 3.36% at 20 suns were achieved for the CIS and CIGS microcells from the nucleation approach, while the CIGS microcells from the LIFT approach reached 0.237% at 20 suns. Heidmann et al. [24] were able to grow CIS micro-absorbers without using patterned substrates by local growth of In islands on Mo-coated glass by PVD, followed by deposition of copper and subsequent selenization, reaching an efficiency of 2.9% under 1 sun and 3.1% at 3 suns. Correia et al. [25] deposited CIS on patterned substrates by electrodeposition, achieving an efficiency of 4.8% under 1 sun. Siopa et al. [26] electrodeposited CIGS absorber layers of 500- and 1500-nanometers-thick into micro-patterned substrates. The microcells demonstrated an increase in efficiencies, reaching 4.8% at 33 suns for the 500-nanometer-thick layer and 5.2% at 3 suns for the 1500-nanometer-thick layer. Table 1 provides a summary of both top-down and bottom-up fabrication approaches for CIGS micro solar cells.

Despite the numerous potential benefits, micro-CPVs also present challenges that need to be addressed. Domínguez et al. [14] have described difficulties related to the components’ size, emphasizing the crucial need for accuracy and precision in manufacturing, manipulation, alignment, and connection of the microcells and micro-optics. Furthermore, the size of these elements can limit the use of specific tools. Achieving large-scale production at low cost requires the development of enabling manufacturing processes already used in the industries for parallel assembly and interconnection of solar cells, such as self-assembly or inkjet printing, along with large-area optical fabrication methods like hot embossing or casting. Additionally, exploring unconventional optical architectures or reconsidering conventional concepts previously discarded due to high material consumption or high bulk absorption at standard CPV might be viable alternatives. An investigation of CIGS micro-CPVs will enable further understanding of the cell materials’ quality, physical and photovoltaic parameters, as well as their operation principles and limitations. This work aims to present a bottom-up approach for the fabrication of pre-structured substrates developed through photolithography and the growth of CIGS micro-absorbers. This study details the process step-by-step, highlighting key parameters and outlining considerations in substrate fabrication. 

## 2. Materials and Methods

Fabrication started with cleaning 1-millimeter-thick soda–lime glass (SLG) substrates using ultrasonic baths with detergent and deionized water. In the first bath, deionized water at room temperature was used with 3 mL of detergent (MicroSon, FisherbrandTM, Fisher Scientific, Landsmeer, The Netherlands) for 10 min. The second bath was performed at 60 °C with detergent for 10 min. From the third to sixth bath, no detergent was used. 

Next, a 500-nanometer-thick Mo back contact bilayer was deposited onto the cleaned SLG substrates by DC magnetron sputtering in the STAR (Sputtering for Advanced Research) sputter-deposition system [27]. To achieve good adhesion of the Mo film to the SLG substrate, a 100-nanometer layer was deposited at 1 Pa pressure, followed by a 400-nanometer layer deposited under a pressure of 0.7 Pa to ensure good conductivity.

### 2.1. Pre-Structured Substrate Fabrication

**Figure 1:**
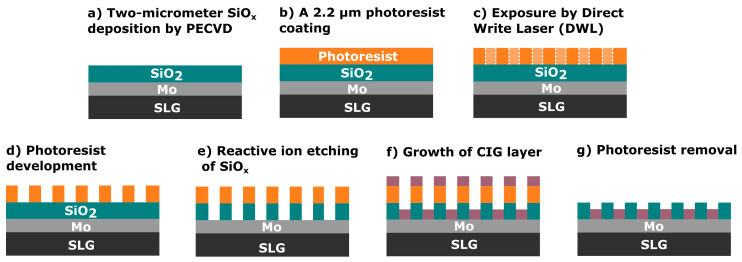
Schematic of the fabrication process of the pre-structured substrates.

The pre-structured substrates (Figure 1) were fabricated in a cleanroom environment, primarily designed for Si wafers, requiring adaptations for using 5 × 5 cm^2^ SLG substrates. The process began with another cleaning step in an ultrasonic bath with acetone, followed by several isopropyl alcohol (IPA) and deionized water rinses. Then, the substrate was vapor-primed in an oven (310TA, Yield Engineering Systems, Inc., Fremont, CA, USA) to remove water and organic residues. 

**Figure 2:**
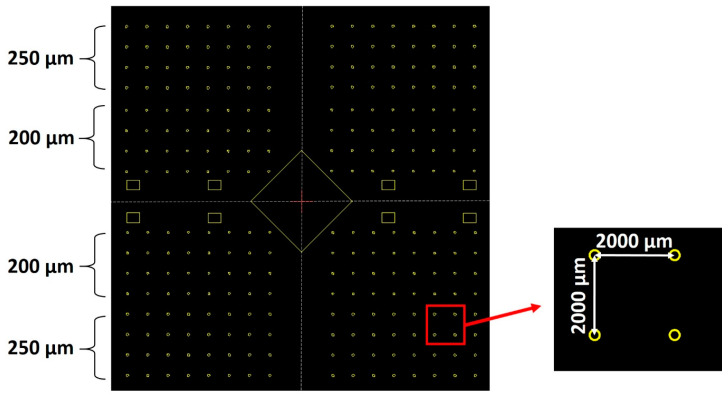
CAD design of the micro-dots pattern matrix. The rectangular shapes represent testing areas for thin film and/or device characterizations.

Next, a 2-micrometer-thick SiO_x_ layer was deposited using plasma-enhanced chemical vapor deposition (MPX CVD Module, SPTS Technologies Ltd., Newport, UK) at 300 °C, with a ~45 nm/min deposition rate. Afterward, the substrate SLG–Mo–SiO_x_ is vapor primed with hexamethyldisilazane (HMDS) in the oven prior to the photoresist coating to enhance adhesion to the substrate. Then, a 2200-nanometer-thick positive photoresist layer (AZ4110, Merck KGaA, Darmstadt, Germany) was spin-coated onto the SiO_x_ layer. The micro-dot pattern was exposed by a direct write laser (DWL 2000, Heidelberg Instruments GmbH, Heidelberg, Germany) lithography system, using a laser power of 120 mW and 405 nm wavelength, followed by the photoresist development (AZ400K 1:4, Merck KGaA, Darmstadt, Germany). Alternatively, a mask aligner (MA6BA6, Suss MicroTec, Garching, Germany) was also used for pattern exposure. The DWL creates high-resolution patterns by laser writing directly onto photoresist-coated samples, whereas the mask aligner requires a physical mask to transfer the pattern. In this case, the mask aligner exposure was performed using a 405 nm wavelength and a low-resolution acetate mask instead of a high-resolution acetate or hard mask. The mask aligner had an exposure time of seconds, whereas, for the DWL, it was at least 10 min. The pattern matrix consisted of circular areas of 200- and 250-micrometer diameters with a 2000-micrometer pitch (Figure 2). Furthermore, a test area of 0.012 mm^2^ was incorporated for characterization purposes.

Subsequently, the micro-holes were etched through the SiO_x_ layer by reactive ion etching (RIE) (APS Module, SPTS, Technologies Ltd., Newport, UK) until reaching the Mo back contact layer, which was confirmed through optical microscope inspections and profilometer measurements. Once the etching is completed, two routes can be considered to finish the substrate and deposit the Cu–In–Ga (CIG) precursor. In the first, employed in this work, a CIG layer was grown by sputtering followed by photoresist lift-off, leaving the SiO_x_ layer with the CIG inside the micro-holes. Subsequently, a plasma asher (M360, PVA TePla America Inc., Corona, CA, USA) was used to remove any residual photoresist. The lifted-off material can be subjected to recovery and recycling processes to produce new targets or to separate the CIG materials [28]. The second route involves area-selective deposition or local growth methods, such as electrodeposition [25,26,29], which are recommended for material saving. In this approach, the photoresist removal is performed after the deposition process.

### 2.2. Structuring the Substrate

**Figure 3:**
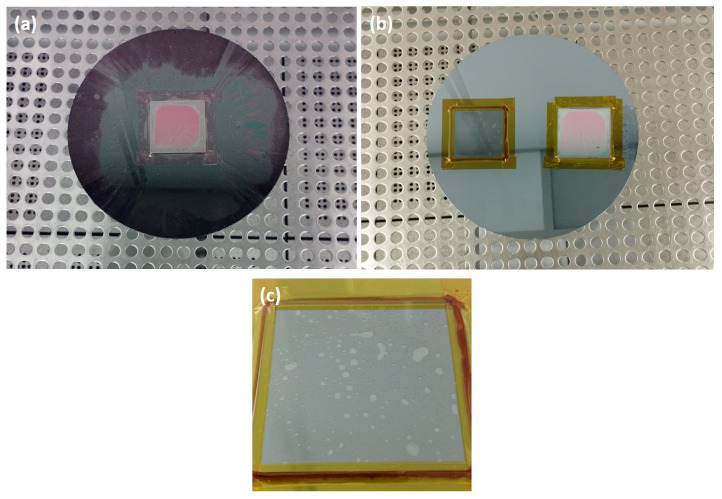
SLG–Mo–SiO_x_ substrate mounted on standard Si wafer used for (**a**) photoresist coating and development; (**b**) SLG–Mo–SiO_x_ substrate mounted next to a bare SLG substrate for RIE; (**c**) close-up of trapped air bubbles in the bare SLG substrate mounted with wax for RIE.

To accommodate the use of 5 × 5 cm^2^ SLG substrates in the cleanroom, where most tools are prepared to handle 8-inch diameter Si wafers, specific sample-mounting adaptations were necessary for the SiO_x_ deposition, photoresist coating and development (Figure 3a), and SiO_x_ etching (Figure 3b,c). 

For photoresist coating, the SLG–Mo–SiO_x_ substrate is fixed on a Si wafer with Kapton tape (Figure 3a). Afterward, for SiO_x_ RIE, a crystalbond wax was used between the Si wafer and the SLG pre-structured substrate for good thermal uniformity. Kapton tape was placed around the substrate to fix it against the Si wafer and maintain the wax (which melts during the SiO_x_ etching process) between the SLG and the Si support wafer (Figure 3b). Also, the pre-structured substrate was mounted on a Si wafer next to an un-processed SLG substrate for proper weight distribution and effective wafer handling in the RIE tool.

### 2.3. CIG Precursor Deposition followed by 2-Stage Selenization

The CIG precursor was deposited from a ternary CIG target (50:35:15 at%, Testbourne) by the magnetron sputtering system STAR [27] at 1.5 W/cm^2^ power density under Ar atmosphere at a working pressure of 0.55 Pa. Subsequently, the resist was removed by lift-off with acetone followed by plasma asher treatment to eliminate any residual photoresist, leaving the SiO_x_ layer with the CIG micro-dots. To prevent Mo oxidation, which starts at 300 °C, a low-temperature plasma asher process was employed.

Thermal annealing was introduced prior to selenization to remove any possible photoresist incorporated into the CIG micro-dot during sputtering [30], to improve the precursor quality. Hence, the sample was placed inside a vacuum pot and placed on a heating plate. To prevent contamination, nitrogen was purged and pumped three times into the pot. Then, the hotplate temperature was set to 500 °C and kept for 30 min once the desired temperature was reached. It is important to note that the temperature inside the vacuum pot was lower than the nominal temperature at the hot plate.

**Figure 4:**
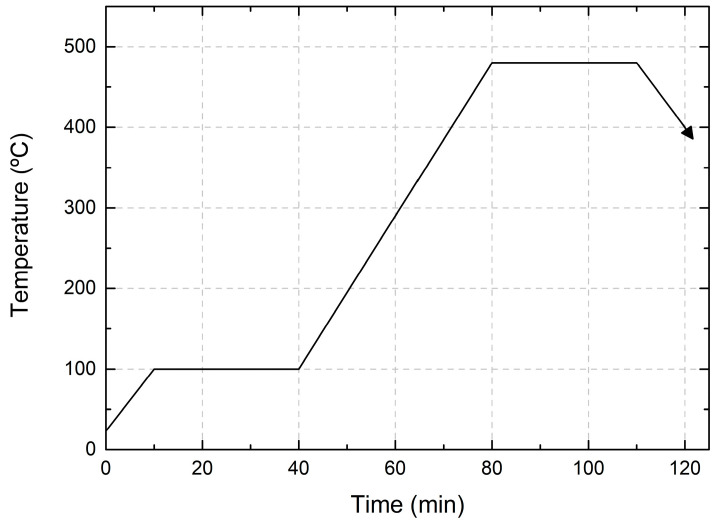
Heating profile for 2-stage selenization.

The CIG precursor was selenized inside a closed graphite box with 50 mg of Se pellets, and loaded inside a quartz tube to obtain the CIGS absorber. The tube underwent a few cycles of nitrogen and argon purging and pumping to minimize the risk of oxygen contamination. During the 2-stage selenization process, in the first stage, the temperature was kept at 100 °C for 30 min to homogenize the precursors. Subsequently, the temperature was increased to 480 °C for 30 min. Once the process was complete, the furnace was cooled down to below 130 °C. A continuous flow of 100 sccm of Ar was maintained throughout the entire process. Furthermore, both heating and cooling ramps were fixed at a slope of 10 and 20 °C/min, respectively. The heating profile of the selenization process is shown in Figure 4. Furthermore, after selenization, a KCN etching (5%) was performed, followed by CdS buffer layer deposition using a chemical bath.

### 2.4. Characterization Methods

The electrical properties of the Mo bilayer were evaluated through 4-point probe measurements before and after the fabrication process to ensure the integrity and functionality of the film. To confirm the dimensions and depth of the micro-holes, optical microscope images and profilometer measurements were conducted during the fabrication process. 

The morphologies and elemental composition analysis of the as-deposited CIG precursor, annealed ones, and CIGS micro-dots were obtained by scanning electron microscopy (SEM) and energy-dispersive X-ray spectroscopy (EDX) in a FEI Quanta 650 FEG (FEI Company, Hillsboro, OR, USA) SEM coupled with an EDX detector. Raman spectroscopy was performed using a confocal Raman microscope (WITec Alpha 300 R, WITec Wissenschaftliche Instrumente und Technologie GmbH, Ulm, Germany) using a 532 nm laser excitation.

## 3. Results and Discussion

### 3.1. Pre-Structured Substrate

To ensure a well-conducting back contact for the CIGS solar cell, the sheet resistance of the Mo bilayer was measured both before and after the fabrication. This measurement also confirms that the SiO_x_ layer was properly etched, reaching the Mo layer. A consistent sheet resistance of (1.5 ± 0.3) Ohm/sq was measured before and after the fabrication, confirming the integrity of the electrical properties of the Mo film.

**Figure 5:**
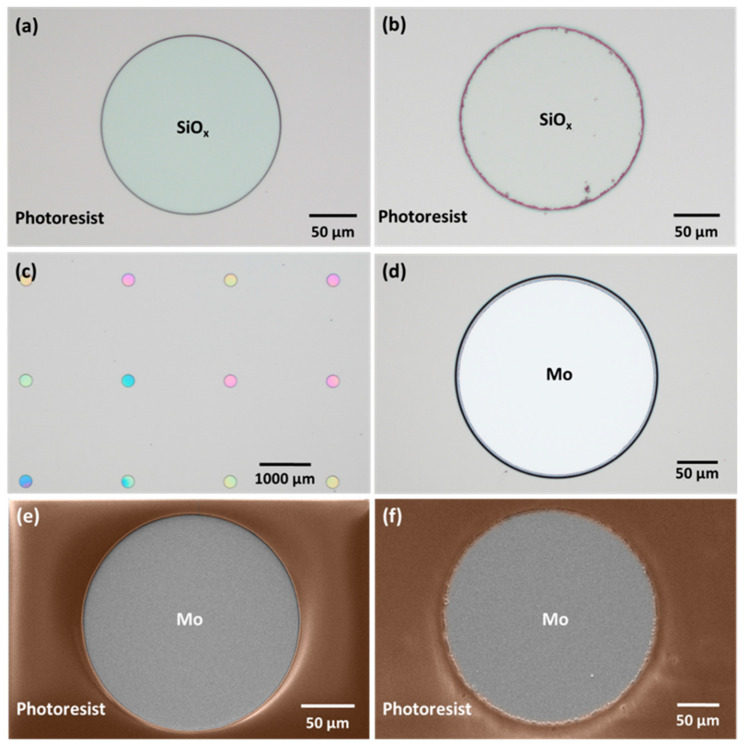
Optical images of micro-holes exposed with (**a**) DWL and (**b**) mask aligner. Micro-holes after (**c**) SiO_x_ etching run (different colors indicate variations in the SiO_x_ layer thickness) and (**d**) completely etched. SEM micrographs of micro-holes exposed with (**e**) DWL and (**f**) mask aligner after SiO_x_ etching.

The choice of tools for the photoresist exposure of the pattern significantly influences the definition and resolution of the micro-holes in the fabrication process. The photoresist exposure using DWL yielded a higher resolution compared to that achieved with a mask aligner, as illustrated in Figure 5a,b. In the latter case, the final pattern’s low resolution can be explained by the intrinsic low resolution of the acetate-printed photomask coupled with the non-optimal contact between the acetate and the glass slide supporting it for mask aligner use. The SEM micrographs show the successful transfer of the mask pattern to the photoresist using DWL (Figure 5e) and the mask aligner (Figure 5f), along with the resulting micro-holes after SiO_x_ etching.

During the RIE process, optical microscopy inspection and profilometer measurements were conducted after each run to verify if the SiO_x_ was completely etched. In Figure 5c, the micro-holes exhibit different colors, indicating slight variations in the SiO_x_ layer thickness after a SiO_x_ etching run. Figure 5d shows a fully etched micro-hole. In some cases, additional SiO_x_ etching runs were necessary to achieve the desired results. While the etching rate of SiO_x_ on standard Si wafers is typically 274 nm/min, on this substrate, the etching rate decreased and became non-uniform, requiring further characterization after each run. This discrepancy could be ascribed to the uneven wax spread during the sample mounting and to trapped air bubbles (Figure 3c), which may lead to thermal non-uniformity and variations in the etching rate. It is crucial to note that the etching process was performed multiple times, with each run lasting a maximum of 4 min, as etching for a longer time can result in the photoresist burning.

### 3.2. CIGS Growth

**Figure 6:**
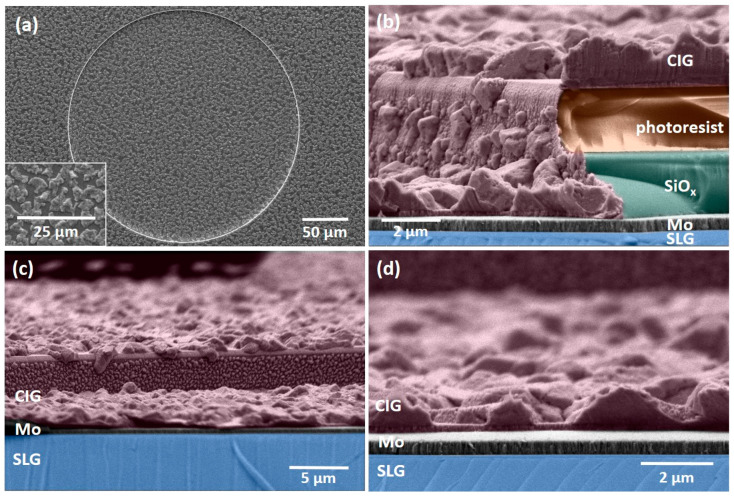
(**a**) Surface SEM micrograph of CIG precursor micro-dot before photoresist removal acquired at 20 kV; (**b**–**d**) cross-sectional SEM micrographs of CIG micro-dot in the SiO_x_–photoresist matrix acquired at 5 kV. Colors were added in post-processing to improve clarity.

Figure 6 shows the surface and cross-sectional micrographs of sputtered CIG precursor micro-dots prior to the photoresist removal. The CIG precursor exhibits a rough surface, with Cu–In-rich grains/islands distributed on the film’s surface. The elemental ratios CGI = [Cu]/([In] + [Ga]) and GGI = [Ga]/([Ga] + [In]) were determined through EDX measurements. The target composition corresponds to the CGI and GGI ratios of 1 and 0.3, respectively. However, due to the sputtering target usage and potential cross-contamination from other targets, these ratios can vary in the deposited films. Consequently, the aimed CIG precursor ratios are defined as 0.7 < CGI < 1 and 0.2 < GGI < 0.3. The precursor film is found to be Cu-poor, with a CGI of 0.88 ± 0.04 and GGI of 0.26 ± 0.02, both within the desired ranges. SEM micrographs and EDX measurements were performed at the same micro-dots after thermal annealing and selenization to obtain reliable data.

**Figure 7:**
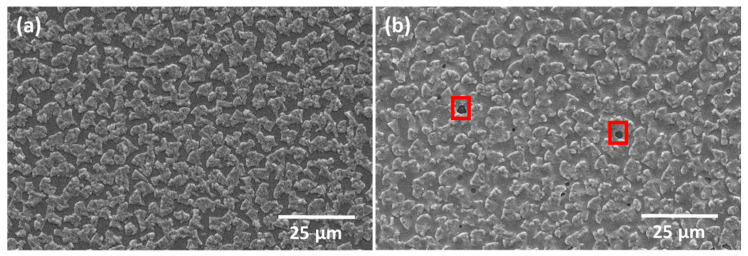
SEM micrographs of (**a**) as-deposited CIG and (**b**) after thermal annealing at 500 °C. Red squares highlight exposed Mo areas.

Figure 7 shows SEM micrographs of the as-deposited CIG film (Figure 7a) and annealed at 500 °C (Figure 7b). In the annealed CIG film, the Cu–In-rich grains appear less distinct compared to the as-deposited CIG, and small areas reveal Mo exposure, which could be related to the degassing of photoresist incorporated during CIG sputtering or debris that was not completely removed after RIE. Notably, the SiO_x_ layer remained intact after the annealing process. The average CGI and GGI ratios for the analyzed micro-dots are 0.84 ± 0.05 and 0.28 ± 0.03, respectively, falling within the expected ranges.

**Figure 8:**
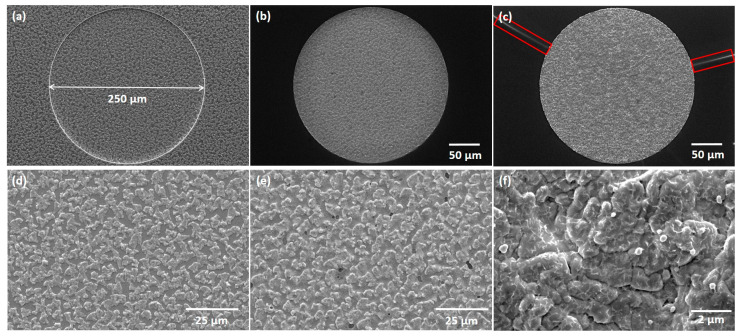
Top-view and up-close SEM micrographs of (**a**–**d**) as-deposited CIG, (**b**–**e**) annealed at 500 °C, and (**c**–**f**) selenized. SEM micrograph (**f**) acquired after CdS deposition. Red rectangles highlight cracking in the SiO_x_ film.

The thermally annealed CIG film underwent a two-stage selenization process at 100 and 480 °C. The SEM micrographs showcase the as-deposited CIG precursor (Figure 8a–d), annealed at 500 °C (Figure 8b–e), and the resulting CIGS micro-dots (Figure 8c–f). The surface of the CIGS micro-dot appears rough and homogenous throughout the micro-dot. It is important to highlight that Figure 8f was acquired following the CdS deposition. A summarized elemental composition is presented in Table 2. The CGI ratio was slightly higher than desired, which can be detrimental to the absorber quality. In high-efficiency solar cells, the CIGS absorber requires a low Ga atomic ratio, approximately 0.2–0.3, and a CGI ratio within the range of 0.88–0.92 [31].

**Figure 9:**
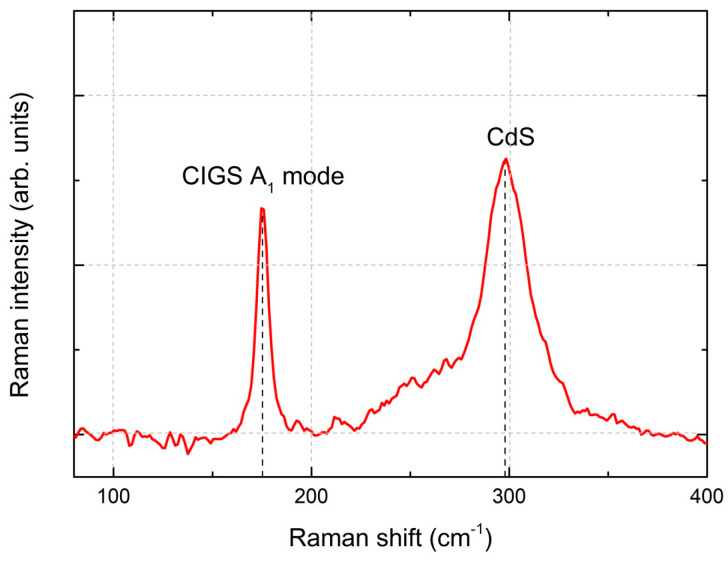
Raman spectrum of CIGS micro-absorber film after KCN (5%) etching and CdS deposition.

In Figure 9, the Raman spectra of a CIGS micro-absorber reveal dominant peaks centered at about 175 and 300 cm^−1^. The peak at 175 cm^−1^ corresponds to the A1 Raman peak of CIGS, associated with the presence of the I-II-VI_2_ chalcopyrite compounds [32]. This peak has a full width at half maximum (FWHM) of 6.5 cm^−1^, indicating a high crystalline quality of the CIGS layer, which is slightly higher but comparable to the FWHM of 3.7 cm^−1^ obtained from a reference single crystal Si sample. Furthermore, the shoulder near 260 cm^−1^ could be attributed to the presence of Cu-Se compounds such as CuSe or Cu_2_Se [32,33]. The peak at 300 cm^−1^ has been identified as the main Raman peak from the CdS buffer layer. After the selenization process, the SiO_x_ layer exhibited some cracking (see red rectangles in Figure 8c), whereas the CIGS micro-dots appeared to remain intact. Given the absence of damage after thermal annealing and in comparable samples after selenization, we hypothesize that mechanical stress incurred during the sample’s breaking into smaller pieces led to cracks during selenization.

## 4. Conclusions

In this study, we present a detailed fabrication process and successfully demonstrate the feasibility of employing a bottom-up approach using pre-structured substrates developed through photolithography for micro-concentrator applications. This approach allows easy modifications to the pattern design for different structures and geometries. 

The choice of method for photoresist exposure significantly influences the resolution and definition of the micro-holes, with a direct-write laser resulting in higher resolution compared to a mask aligner. The use of a high-resolution acetate or hard mask should improve the exposure resolution but in both cases no modifications to the pattern designs are possible. 

While the fabrication process required some adaptations, the major challenge was encountered during the SiO_x_ etching process, which required intensive inspection after each run and sometimes multiple etching runs to achieve the desired depth to reach the Mo layer. 

CIGS micro-absorbers were prepared via sputtering of the CIG precursor into a patterned SiO_x_ matrix, followed by lift-off, thermal annealing at 500 °C, and a two-stage selenization process. The results indicate that both the as-deposited and annealed CIG films were Cu-poor, with CGI ratios of 0.88 and 0.84, respectively. The surface of the as-deposited CIG film exhibited roughness, characterized by CIG grains throughout the film. Subsequent thermal annealing at 500 °C led to less pronounced grains. After the two-stage selenization, the CIGS film had a CGI ratio of 1 and exhibited Raman spectra with a dominant A1 mode peak at 175 cm^−1^, observed in I-III-VI_2_ chalcopyrite compounds. In future work, we aim to improve the CIGS absorber material by optimizing the two-stage selenization temperature and to produce complete CIGS microcells by deposing buffer and window layers. 

## Figures and Tables

**Table 1:** Summary of top-down and bottom-up fabrication approaches of CIGS micro solar cells.

Fabrication	Deposition Method	Material	Size of Micro Solar Cell (µm)	Efficiency under 1 Sun (%)	Efficiency under Concentration (%)	Reference
**Top-Down**						
Photolithography after buffer	Co-evaporation	Cu(In,Ga)Se_2_	15	13	17 (×120)	[19]
Photolithography after i-ZnO	Co-evaporation	Cu(In,Ga)Se_2_	15	~14	~17	[18]
			25	~11.5	~16.5 (×120)	
			50	~13	~16.5	
Photolithography after i-ZnO	Co-evaporation	Cu(In,Ga)Se_2_	50	16.3	21.3 (×475)	[15]
Photolithography and chemical etching	Co-evaporation	Cu(In,Ga)Se_2_	40	~13	~17 (×100)	[21]
			250	~11.5	~15 (×40)	
P1, P2, and P3 scribing	Co-evaporation	Cu(In,Ga)Se_2_	1000 *	13.5	15 (×6)	[22]
			500 *	11.8	14.6 (×8)	
			750 (dot-shaped)	13.6	17.6 (×51)	
**Bottom-Up**						
Patterned substrate	PVD	CuInSe_2_	40–60	2.9	3.1 (×3)	[24]
Laser-patterned substrate	Nucleation (PVD)	CuInSe_2_ Cu(In,Ga)Se_2_	~400	2.9	3.06 (×3)	[23]
				1.4	3.36 (×20)	
Laser-patterned substrate	LIFT	Cu(In,Ga)Se_2_	~490	0.15	0.237 (×20)	[23]
Patterned substrate	Area-selective electrodeposition	CuInSe_2_	200	4.8	-	[25]
Patterned substrate	Area-selective electrodeposition	Cu(In,Ga)Se_2_	200	4.8	5.2 (×3)	[26]

* lateral cell width.

**Table 2:** Elemental compositions of the as-deposited, annealed, and CIGS films.

Sample	Composition (at %)				Ratios	
	Cu	In	Ga	Se	CGI	GGI
CIG	46 ± 1.9	38 ± 1.8	13 ± 1.0	-	0.88 ± 0.04	0.26 ± 0.02
CIG500	45 ± 1.7	39 ± 1.7	15 ± 1.8	-	0.84 ± 0.04	0.28 ± 0.03
CIGS	24 ± 0.8	20 ± 1.5	5 ± 1.5	50 ± 0.9	1.00 ± 0.04	0.20 ± 0.06

## Data Availability

The data presented in this study are available on request from the corresponding author.

## References

[1] Masson G., Bosch E., van Rechem A., de I’Epine M., Kaizuka I., Jäger-Waldau A., Donoso J. (2023). Snapshot of Global PV Markets 2023.

[2] Feldman D., Zuboy J., Dummit K., Stright D., Heine M., Mirletz H., Margolis R. (2024). Winter 2024 Solar Industry Update.

[3] Philipps S., Warmuth W., Bett A.W., Burger B., Friedrich L., Kost C., Nold S., Peper D., Preu R., Rentsch J. Photovoltaics Report. https://www.ise.fraunhofer.de/content/dam/ise/de/documents/publications/studies/Photovoltaics-Report.pdf.

[4] Slade A., Garboushian V. 27.6% Efficient Silicon Concentrator Solar Cells for MassProduction. Proceedings of the 15th International Photovoltaic Science and Engineering Conference.

[5] Green M.A., Dunlop E.D., Yoshita M., Kopidakis N., Bothe K., Siefer G., Hao X. (2023). Solar Cell Efficiency Tables (Version 62). Prog. Photovoltaics Res. Appl..

[6] Kashyap S., Madan J., Pandey R., Sharma R. (2020). Comprehensive Study on the Recent Development of PERC Solar Cell. Conf. Rec. IEEE Photovolt. Spec. Conf..

[7] Keller J., Kiselman K., Donzel-Gargand O., Martin N.M., Babucci M., Lundberg O., Wallin E., Stolt L., Edoff M. (2024). High-Concentration Silver Alloying and Steep Back-Contact Gallium Grading Enabling Copper Indium Gallium Selenide Solar Cell with 23.6% Efficiency. Nat. Energy.

[8] Ward J.S., Egaas B., Noufi R., Contreras M., Ramanathan K., Osterwald C., Emery K. Cu(In,Ga)Se_2_ Solar Cells Measured under Low Flux Optical Concentration. Proceedings of the 2014 IEEE 40th Photovoltaic Specialist Conference (PVSC).

[9] Khamooshi M., Salati H., Egelioglu F., Hooshyar Faghiri A., Tarabishi J., Babadi S. (2014). A Review of Solar Photovoltaic Concentrators. Int. J. Photoenergy.

[10] Wiesenfarth M., Anton I., Bett A.W. (2018). Challenges in the Design of Concentrator Photovoltaic (CPV) Modules to Achieve Highest Efficiencies. Appl. Phys. Rev..

[11] Dimroth F., Tibbits T.N.D., Niemeyer M., Predan F., Beutel P., Karcher C., Oliva E., Siefer G., Lackner D., Fus-Kailuweit P. (2016). Four-Junction Wafer-Bonded Concentrator Solar Cells. IEEE J. Photovoltaics.

[12] Kayes B.M., Zhang L., Twist R., Ding I.K., Higashi G.S. (2014). Flexible Thin-Film Tandem Solar Cells with >30% Efficiency. IEEE J. Photovoltaics.

[13] Paire M., Lombez L., Guillemoles J.F., Lincot D. (2010). Toward Microscale Cu(In,Ga)Se_2_ Solar Cells for Efficient Conversion and Optimized Material Usage: Theoretical Evaluation. J. Appl. Phys..

[14] Domínguez C., Jost N., Askins S., Victoria M., Antón I. (2017). A Review of the Promises and Challenges of Micro-Concentrator Photovoltaics. AIP Conf. Proc..

[15] Paire M., Lombez L., Donsanti F., Jubault M., Lincot D., Guillemoles J.-F., Collin S., Pelouard J.-L. (2013). Thin-Film Microcells: A New Generation of Photovoltaic Devices. SPIE Newsroom.

[16] Alves M., Pérez-Rodríguez A., Dale P.J., Domínguez C., Sadewasser S. (2020). Thin-Film Micro-Concentrator Solar Cells. J. Phys. Energy.

[17] Sadewasser S., Salomé P.M.P., Rodriguez-Alvarez H. (2017). Materials Efficient Deposition and Heat Management of CuInSe_2_ Micro-Concentrator Solar Cells. Sol. Energy Mater. Sol. Cells.

[18] Paire M., Shams A., Lombez L., Péré-Laperne N., Collin S., Pelouard J.L., Guillemoles J.F., Lincot D. (2011). Resistive and Thermal Scale Effects for Cu(In,Ga)Se_2_ Polycrystalline Thin Film Microcells under Concentration. Energy Environ. Sci..

[19] Paire M., Lombez L., Péré-Laperne N., Collin S., Pelouard J.-L., Lincot D., Guillemoles J.-F. (2011). Microscale Solar Cells for High Concentration on Polycrystalline Cu(In,Ga)Se_2_ Thin Films. Appl. Phys. Lett..

[20] Paire M., Lombez L., Donsanti F., Jubault M., Collin S., Pelouard J.L., Guillemoles J.F., Lincot D. (2013). Cu(In, Ga)Se_2_ Microcells: High Efficiency and Low Material Consumption. J. Renew. Sustain. Energy.

[21] Paire M., Jean C., Lombez L., Collin S., Pelouard J.-L., Gérard I., Guillemoles J.-F., Lincot D. (2015). Cu(In,Ga)Se_2_ Mesa Diodes for the Study of Edge Recombination. Thin Solid Films.

[22] Reinhold B., Schmid M., Greiner D., Schüle M., Kieven D., Ennaoui A., Lux-steiner M.C. (2015). Monolithically Interconnected Lamellar Cu(In,Ga)Se_2_ Micro Solar Cells under Full White Light Concentration. Prog. Photovoltaics Res. Appl..

[23] Ringleb F., Andree S., Heidmann B., Bonse J., Eylers K., Ernst O., Boeck T., Schmid M., Krüger J. (2018). Femtosecond Laser-Assisted Fabrication of Chalcopyrite Micro-Concentrator Photovoltaics. Beilstein J. Nanotechnol..

[24] Heidmann B., Ringleb F., Eylers K., Levcenco S., Bonse J., Andree S., Krüger J., Unold T., Boeck T., Lux-Steiner M.C. (2017). Local Growth of CuInSe_2_ Micro Solar Cells for Concentrator Application. Mater. Today Energy.

[25] Correia D., Siopa D., Colombara D., Tombolato S., Salomé P.M.P., Abderrafi K., Anacleto P., Dale P.J., Sadewasser S. (2019). Area-Selective Electrodeposition of Micro Islands for CuInSe_2_-Based Photovoltaics. Results Phys..

[26] Siopa D., El Hajraoui K., Tombolato S., Babbe F., Lomuscio A., Wolter M.H., Anacleto P., Abderrafi K., Deepak F.L., Sadewasser S. (2020). Micro-Sized Thin-Film Solar Cells via Area-Selective Electrochemical Deposition for Concentrator Photovoltaics Application. Sci. Rep..

[27] Fuster D., Anacleto P., Virtuoso J., Zutter M., Brito D., Alves M., Aparicio L., Fuertes Marrón D., Briones F., Sadewasser S. (2020). System for Manufacturing Complete Cu(In,Ga)Se_2_ Solar Cells in Situ under Vacuum. Sol. Energy.

[28] Hu D., Ma B., Li X., Lv Y., Chen Y., Wang C. (2022). Innovative and Sustainable Separation and Recovery of Valuable Metals in Spent CIGS Materials. J. Clean. Prod..

[29] Duchatelet A., Nguyen K., Grand P.P., Lincot D., Paire M. (2016). Self-Aligned Growth of Thin Film Cu(In,Ga)Se_2_ Solar Cells on Various Micropatterns. Appl. Phys. Lett..

[30] Poeira R.G., Pérez-Rodríguez A., Prot A.J.M., Alves M., Dale P.J., Sadewasser S. (2023). Direct Fabrication of Arrays of Cu(In,Ga)Se_2_ Micro Solar Cells by Sputtering for Micro-Concentrator Photovoltaics. Mater. Des..

[31] Ramanujam J., Singh U.P. (2017). Copper Indium Gallium Selenide Based Solar Cells—A Review. Energy Environ. Sci..

[32] Zaretskaya E.P., Gremenok V.F., Riede V., Schmitz W., Bente K., Zalesski V.B., Ermakov O.V. (2003). Raman Spectroscopy of CuInSe_2_ Thin Films Prepared by Selenization. J. Phys. Chem. Solids.

[33] Choi I.H. (2011). Raman Spectroscopy of CuIn_1−X_Ga_x_Se_2_ for in-Situ Monitoring of the Composition Ratio. Thin Solid Films.

